# Urinary tract infection in outpatients in Germany: a cross-sectional study of diagnostics and susceptibility testing in medical laboratories

**DOI:** 10.3399/BJGPO.2025.0004

**Published:** 2025-12-19

**Authors:** Hannah Bender, Kathrin Jobski, Guido Schmiemann, Axel Hamprecht, Falk Hoffmann

**Affiliations:** 1 Department of Health Services Research, Carl von Ossietzky Universität Oldenburg, Oldenburg, Germany; 2 Department of Health Services Research, University Bremen, Bremen, Germany; 3 Institute of Medical Microbiology and Virology, Carl von Ossietzky Universität Oldenburg, Oldenburg, Germany

**Keywords:** urinary tract infections, outpatients, antimicrobial stewardship, guideline adherence, health services research, primary health care

## Abstract

**Background:**

Urinary tract infections (UTIs) are common, representing a frequent cause of antibiotic prescription in primary care worldwide. Selection of antibiotics for antimicrobial susceptibility testing and the reporting of test results by laboratories can directly impact antibiotic prescribing and guideline adherence.

**Aim:**

To assess the current practice of susceptibility testing by laboratories for outpatient UTIs in Germany.

**Design & setting:**

A cross-sectional study was conducted including all laboratories identified by searching for specialists in laboratory medicine and microbiology on the websites of the 17 German associations of statutory health insurance physicians.

**Method:**

Between January and April 2024, a survey using a standardised questionnaire was conducted across identified laboratories.

**Results:**

Of the 396 laboratories identified, 65.2% (*n* = 258) replied. Of these, 106 laboratories performed susceptibility testing and on average tested for 13.1 (standard deviation [SD] 3.6) different antibiotics in a urine culture positive for *Escherichia coli*. The most commonly tested antibiotics were ciprofloxacin (98.1%), co-trimoxazole (97.2%), cefuroxime, and nitrofurantoin (both 91.5%). On average, laboratories tested 3.8 of the five antibiotics recommended in the German guidelines on uncomplicated UTI, with 26.4% testing for all five. Laboratories received clinical information on previous treatments and comorbidities in an estimated one-fifth (on average 21.3% and 21.5%, respectively) of the urine samples, and information on the type of the urine sample in an estimated three-fifths (63.7%) of samples.

**Conclusion:**

Laboratories should test and report as many first-line antibiotics as possible. Further, a more detailed and standardised transfer of clinical information to laboratories could enhance the quality of antibiotic prescribing.

## How this fits in

This study provides novel insights into the current practice of antimicrobial susceptibility testing for outpatient urinary tract infections (UTIs) in Germany. Current susceptibility testing for outpatient UTIs in Germany varies, with 84.9% of laboratories testing for ≥3 first-line antibiotics while 15.1% test for ≤2 antibiotics. Laboratories receive clinical information about previous treatments and comorbidities in only about 20% of the urine samples. Standardising clinical information transfer between outpatient physicians and laboratories could improve guideline adherence and antibiotic selection.

## Introduction

Urinary tract infections (UTIs) are among the most common infections and represent a frequent cause of medical consultations in primary care.^1^ More than 150 million people worldwide are affected by community-acquired UTIs each year.^2^ Although UTIs can be self-limiting, they are a common reason for antibiotic prescribing.^1,3,4^

As antibiotic resistance is increasing worldwide, particularly among gram-negative bacteria, it is important to avoid its inappropriate use.^5,6^ However, several studies have shown that treatment of UTIs in the outpatient setting often does not follow guideline recommendations, which can lead to inappropriate antibiotic use.^4,7,8^ Although Malmros *et al* identified differences in the guidelines across 15 European countries, 12 of the 15 guidelines recommended nitrofurantoin as the first-line treatment for uncomplicated UTIs, followed by pivmecillinam and fosfomycin, which were listed second and third most frequently.^9^ The two previous German guidelines, as well as the latest interdisciplinary guideline, recommend as first-line treatment for uncomplicated UTIs fosfomycin trometamol, nitrofurantoin, nitroxoline, and pivmecillinam. In case of local resistance rates of <20% trimethoprim can be equally considered.^10,11^ Moreover, the guidelines recommend against the use of fluoroquinolones and cephalosporins as first-line treatment for outpatient UTIs.^10–12^

In selecting an appropriate antibiotic, a urine culture can be a useful tool. The German guideline on uncomplicated UTI recommends urine cultures in cases of ambiguous symptoms, or recurrent or complicated UTIs.^12^ The interpretation of a urine culture result not only depends on bacterial count and the pathogen detected, but also on the type of urine collection, the presence or absence of leukocyturia, and other clinical information from the patient.^13^ In instances where a urine culture is conducted and typical uropathogens are identified, the laboratory performs susceptibility testing for a pre-defined range and number of antibiotics.^14^

Studies show that the reporting of susceptibility testing varies, which can directly impact antibiotic prescribing and guideline adherence in the treatment of UTIs.^15–18^ An earlier German study found that microbiological tests often did not include the first-line antibiotics recommended in the guidelines.^14^ However, this study was conducted >10 years ago and covered only northern Germany.^14^ Furthermore, the exchange between laboratories and primary care is less well studied.

Therefore, the aim of this cross-sectional study was two-fold, to describe a) the current practice of susceptibility testing by laboratories for outpatient UTIs in Germany, including the range of antibiotics tested; and b) the communication and exchange of information between outpatient physicians and laboratories.

## Method

### Study design and study population

A cross-sectional study was conducted among medical laboratories in Germany using a standardised questionnaire (data collected January–April 2024). As no list of all microbiology laboratories in Germany was available, laboratories were identified by searching the websites of all 17 German associations of statutory health insurance (SHI) physicians for all registered specialists in the two relevant medical specialties (microbiology, virology, and infectious epidemiology, as well as laboratory medicine). Using their registered addresses, we identified the corresponding laboratories and determined contact details via internet research. In two of the 17 associations (that is, Bavaria and Rhineland-Palatinate) no search via their websites was offered. In the case of Bavaria, the laboratories were identified via the Bavarian Medical Association, and in the case of Rhineland-Palatinate, via the website of the Federal Ministry of Health (https://gesund.bund.de). In addition, the list of laboratories was compared with the list of accredited laboratories (Deutsche Akkreditierungsstelle) and supplemented where necessary.

Participation in the survey was possible by telephone, email, or fax. The questionnaire could be completed by medical or laboratory staff. In a first step, laboratories were contacted by telephone. During the initial telephone call, the interviewer provided a brief explanation of the survey’s content and requested to be connected with medical staff or other appropriately qualified laboratory staff. It was possible to take part in the survey directly, to arrange a telephone appointment, or to receive further information and the questionnaire by email or fax. When participating by telephone, the interviewer filled out the questionnaire. If a laboratory could not be reached after five attempts by telephone at different times, it was contacted by email. If an email was sent successfully, no further contact was made.

The methodological approach (identifying laboratories, contacting them, and piloting of the questionnaire) was pre-tested in two federal German states (that is, Bremen and Lower Saxony). The pre-test led to a change in the method of initial contact (that is, from fax to telephone).

### Instrument and included variables

The questionnaire consisted of eight items. Initially, besides the federal state, the laboratory was asked if it performed susceptibility testing for outpatient UTI. If yes, the laboratory was asked which testing standards were used. Answers included the Clinical and Laboratory Standards Institute, the European Committee on Antimicrobial Susceptibility Testing (EUCAST), and the National Antimicrobial Susceptibility Testing Committee. Responders could provide a free-text response and multiple responses were allowed. As *Escherichia coli (E. coli*) is clearly the most common pathogen causing uncomplicated and complicated UTIs, laboratories were asked to report on their practice of susceptibility testing for this species.^19^ Additionally, the bacterial count (in colony-forming units [CFU]/ml), which prompts testing of *E. coli* in pure culture, was requested (options '10² CFU/ml', '10³ CFU/ml', '10⁴ CFU/ml', and 'other').^10,11^ Laboratories were also asked to list all antibiotics that are routinely tested for susceptibility for *E. coli*. The questionnaire listed 14 antibiotics (amoxicillin, amoxicillin and clavulanic acid, cefpodoxime, cefuroxime, ciprofloxacin, co-trimoxazole, fosfomycin, levofloxacin, nitrofurantoin, nitroxoline, norfloxacin, ofloxacin, pivmecillinam, and trimethoprim) in alphabetical order, including those recommended in the previous German guidelines,^10,11^ in the quality standards (Mikrobiologisch-infektiologische Qualitätsstandards [MIQ]),^13^ and a group of frequently used antibiotics.^4^ Laboratories could also list any additional substances tested. Subsequently, situations were asked in which susceptibility tests are conducted for antibiotics not previously mentioned or for a different number of substances, and it was possible to explain these situations in more detail. In addition, participants were asked which information (besides age and sex) they would like to receive with the urine samples. Lastly, the proportion of urine samples with accompanying information (type of urine collection, antibiotic pre-treatment, and comorbidities) should be estimated.

### Statistical analysis

Descriptive statistics (mean, standard deviation [SD], median, and percentages) were used. Inductive categorisation was performed to summarise the open-ended questions. Participants' responses were categorised and quantified by two independent persons (HB and KJ). If there was a disagreement, a third person was consulted. Denominators vary depending on the question. SPSS Statistics for iOS (version 28.0.1.0) was used for all statistical analyses.

## Results

### Baseline characteristics of the study population

**Figure 1.**
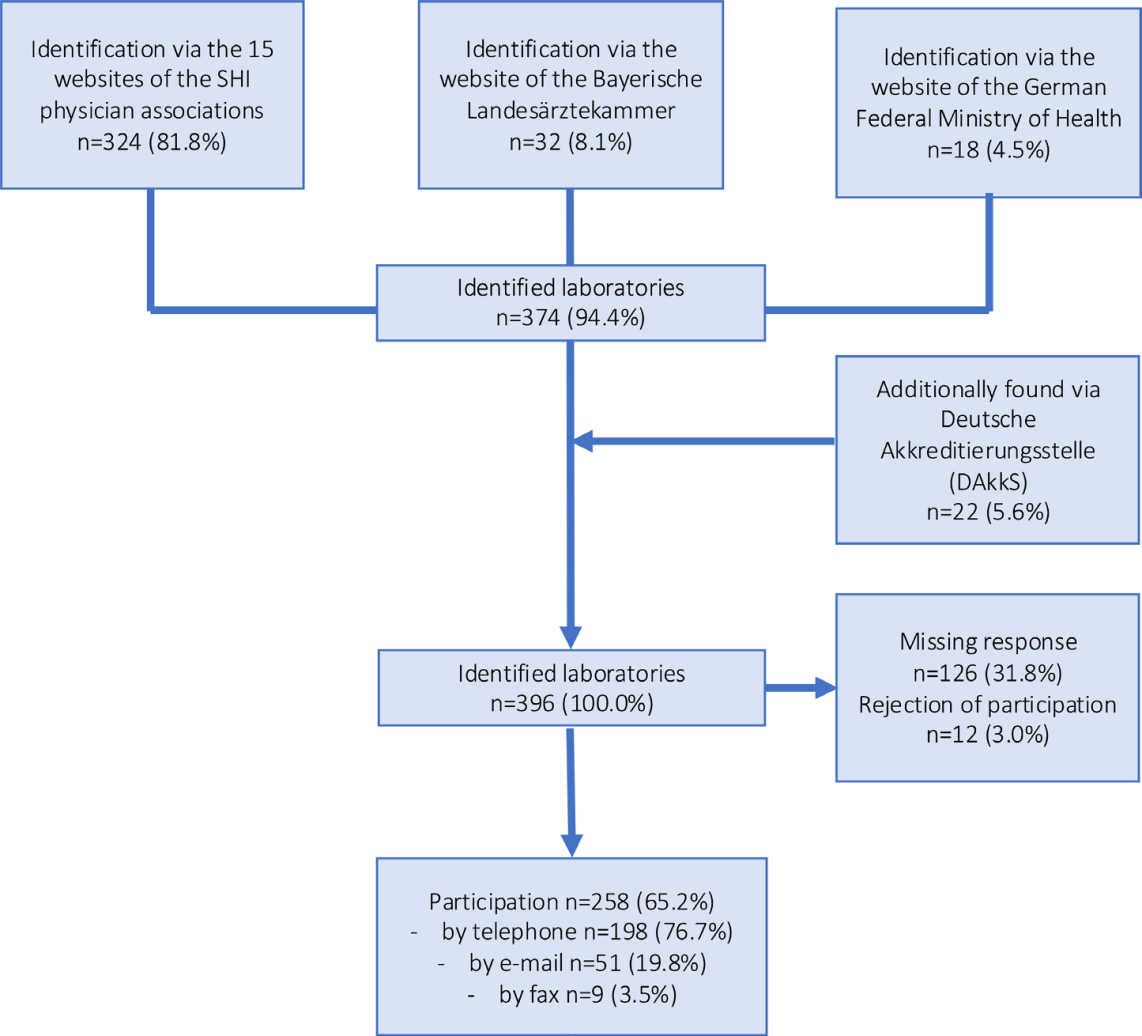
Flowchart of the survey process. SHI = statutory health insurance.

A total of 396 laboratories were identified (Figure 1). Of these, 65.2% (*n* = 258 laboratories) provided information on whether or not they perform susceptibility testing in outpatient UTIs, with 76.7% (*n* = 198) of these responding via telephone. A total of 106 laboratories performed susceptibility testing for outpatient UTI (Table 1).

**Table 1.** Baseline characteristics of the study population

**Characteristic**	**Total, *N* = 258, *n* (%)**	**Performing susceptibility tests, *n* (%)**	
		**Yes, *n* = 106**	**No, *n* = 152**
**Federal state^a^**	**253**	**101**	**152**
Baden-Wuerttemberg	11 (4.3)	9 (8.9)	2 (1.3)
Bavaria	20 (7.9)	13 (12.9)	7 (4.6)
Berlin	10 (4.0)	1 (1.0)	9 (5.9)
Brandenburg	17 (6.7)	4 (4.0)	13 (8.6)
Hamburg	12 (4.7)	5 (5.0)	7 (4.6)
Hesse	21 (8.3)	8 (7.9)	13 (8.6)
Lower Saxony	27 (10.7)	12 (11.9)	15 (9.9)
Mecklenburg-Western Pomerania	19 (7.5)	5 (5.0)	14 (9.2)
North Rhine-Westphalia	52 (20.6)	20 (19.8)	32 (21.1)
Rhineland-Palatinate	13 (5.1)	4 (4.0)	9 (5.9)
Saarland	4 (1.6)	2 (2.0)	2 (1.3)
Saxony	20 (7.9)	8 (7.9)	12 (7.9)
Saxony-Anhalt	11 (4.3)	5 (5.0)	6 (3.9)
Schleswig-Holstein	7 (2.8)	2 (2.0)	5 (3.3)
Thuringia	9 (3.6)	3 (3.0)	6 (3.9)
**Participation by medium**	**258**	**106**	**152**
Telephone	198 (76.7)	48 (45.3)	150 (98.7)
Email	51 (19.8)	49 (46.2)	2 (1.3)
Fax	9 (3.5)	9 (8.5)	0 (0.0)

^a^ *n* = 5 practices did not provide federal state.

### Test standards and bacterial count limit used

Overall, 101 (96.2%) laboratories performed the antibiotic susceptibility testing according to EUCAST (Table 2). A total of 50.9% (*n* = 54) of laboratories performed susceptibility tests for *E. coli* in pure culture with a bacterial count of 10^3^ CFU/ml. A total of 19.8% (*n* = 21) and 4.7% (*n* = 5) of the laboratories carried out susceptibility tests with a bacterial count of 10^4^ CFU/ml and ≥10^5^ CFU/ml, respectively. In total, 8.5% of laboratories reported that factors such as inhibitor test results and the presence of leukocytes influence the bacterial count limit used to decide when to perform antimicrobial susceptibility testing.

**Table 2.** Characteristics of laboratories performing susceptibility testing for outpatient urinary tract infections

Characteristic	*n* (%)^a^
Testing standard used, *n* = 105^b^	
Clinical and Laboratory Standards Institute	5 (4.8)
European Committee on Antimicrobial Susceptibility Testing	101 (96.2)
National Antimicrobial Susceptibility Testing Committee	59 (56.2)
Other	1 (1.0)
**Bacterial count limit used, *n* = 106** ^**b**^	
10² CFU/ml	16 (15.1)
10³ CFU/ml	54 (50.9)
10⁴ CFU/ml	21 (19.8)
Use of bacterial count ≥10^5^ CFU/ml	5 (4.7)
Bacterial count limit based on inhibiting test	5 (4.7)
Bacterial count limit based on detection of leukocytes	4 (3.8)
Bacterial count limit based on the type or number of pathogens that are detected	3 (2.8)
Bacterial count limit based on the sender’s requirements	2 (1.9)
**Deviation from the previously listed standard of susceptibility tests, *n* = 104**	
Yes	84 (80.8)
No	20 (19.2)
**Description of situations in which deviating susceptibility tests are performed, *n* = 82^b^**	
Detection of (multidrug-)resistant pathogens	56 (68.3)
Multidrug-resistant gram-negative bacteria	31 (37.8)
Detection of other pathogens	8 (9.8)
Sender request	26 (31.7)
Special patient groups	11 (13.4)
Inpatients	5 (6.1)
Children	3 (3.7)
**Desired information accompanying the urine sample, *n* = 101** ^**b**^	
Clinical information or special features	57 (56.4)
Pregnancy	13 (12.9)
Immunosuppression	8 (7.9)
Antibiotic (pre)treatment	56 (55.4)
Information on the material or type of urine sample	46 (45.5)
Catheter urine	20 (19.8)
Diagnosis or suspected diagnosis	29 (28.7)
Leukocyte detection or leukocyturia	19 (18.8)
Exact date of urine collection	8 (7.9)
Other	4 (4.0)
**Estimated proportion of urine samples with indication of the type of urine, *n* = 99**	
Mean (SD)	63.7 (37.0)
Median (IQR)	80.0 (20.0–95.0)
**Estimated proportion of urine samples with indication of pretreatment, *n* = 96**	
Mean (SD)	21.3 (23.4)
Median (IQR)	10.0 (5.0–30.0)
**Estimated proportion of urine samples with information on concomitant diseases, *n* = 100**	
Mean (SD)	21.5 (24.5)
Median (IQR)	10.0 (5.0–30.0)

^a^Unless otherwise stated. ^b^Multiple answers possible. IQR = interquartile range. SD = standard deviation.

### Antibiotics tested

**Figure 2.**
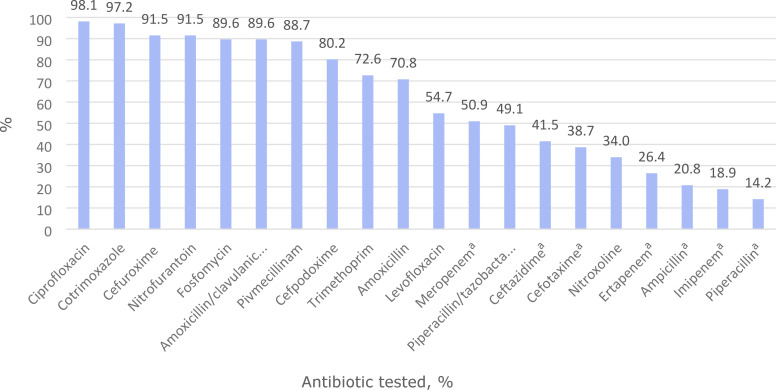
List of the 20 most frequently tested antibiotics. Antibiotics tested, *n* = 106. ^a^Additional antibiotic not listed in the questionnaire.

On average, laboratories performed susceptibility tests for 13.1 (SD 3.6) antibiotics (Table 3). Laboratories most commonly tested for ciprofloxacin (98.1%), co-trimoxazole (97.2%), cefuroxime, and nitrofurantoin (both 91.5%), while 72.6% tested for trimethoprim and 34.0% for nitroxoline (Figure 2). Susceptibility testing of substances not previously listed in the questionnaire was reported by 67.9% of laboratories, with meropenem (50.9%), piperacillin and tazobactam (49.1%), ceftazidime (41.5%), and cefotaxime (38.7%) being the most common antibiotics.

**Table 3.** Antibiotic testing for outpatient urinary tract infections in German laboratories (*N* = 106)^10,11^

Category	***n* (%)^a^**
Number of antibiotics tested per laboratory	
6–9 antibiotics tested	16 (15.1)
10–13 antibiotics tested	40 (37.7)
14–17 antibiotics tested	40 (37.7)
≥18 antibiotics tested	10 (9.4)
Mean (SD)	13.1 (3.6)
Median (IQR)	13.0 (11.0–15.0)
**Number of antibiotics tested per laboratory recommended in the guidelines^b^**	
0 recommended antibiotics tested	1 (0.9)
1 recommended antibiotic tested	4 (3.8)
2 recommended antibiotics tested	11 (10.4)
3 recommended antibiotics tested	15 (14.2)
4 recommended antibiotics tested	47 (44.3)
5 recommended antibiotics tested	28 (26.4)
Mean (SD)	3.8 (1.1)
Median (IQR)	4.0 (3.0–5.0)

^a^Unless otherwise stated.^ b^The two valid guidelines^10,11^ at the time of data collection recommend as first-line treatment for uncomplicated urinary tract infections fosfomycin trometamol, nitrofurantoin, nitroxoline, pivmecillinam, and trimethoprim, with local resistance rates of <20%. IQR = interquartile range. SD = standard deviation.

The majority of laboratories (80.8%, *n* = 84) reported performing susceptibility testing for different antibiotics in specific cases. In particular, 68.3% (*n* = 56) reported performing such tests for (multidrug)-resistant pathogens. In addition, 31.7% (*n* = 26) reported performing susceptibility testing at the request of the sender, 13.4% (*n* = 11) reported doing so for specific patient groups (for example, hospitalised patients or children), while 9.8% (*n* = 8) reported performing these tests to detect other pathogens (Table 2).

On average, laboratories tested for 3.8 (SD 1.1) of the five antibiotics recommended in the recent guidelines, with only 26.4% (*n* = 28) of laboratories testing for all (Table 3). A total of 84.9% (*n* = 90) of laboratories tested for ≥3 first-line antibiotics, while 15.1% (*n* = 16) tested ≤2.

### Information accompanying the urine sample

A total of 101 laboratories provided information on the desired accompanying information for urine samples (Table 2). Of those, 56.4% (*n* = 57) desired detailed clinical information, particularly information about pregnancy (12.9%, *n* = 13) or immunosuppression (7.9%, *n* = 8). In addition, 55.4% (*n* = 56) stated that they needed information about current or previous antibiotic therapy. Further, 45.5% considered precise information on the type of urine sample, particularly information on catheter urine (19.8%, *n* = 20), to be particularly relevant. In addition, 28.7% (*n* = 29) considered it necessary to provide a diagnosis or suspected diagnosis and 18.8% (*n* = 19) to indicate results of any urine dipstick examination and evidence of leukocyturia.

The participants estimated that, on average, they received information on the exact type of urine sample in 63.7% (SD 37.0) of the cases. With regard to possible previous treatment or existing concomitant diseases, information was estimated to be available for a mean proportion of only 21.3% (SD 23.4) and 21.5% (SD 24.5) of the urine samples, respectively.

## Discussion

### Summary

In our cross-sectional study, we found that only 26.4% of German laboratories tested for all five antibiotics recommended in the guidelines for uncomplicated UTI, with trimethoprim (72.6%) and nitroxoline (34.0%) being tested the least. The most frequently tested antibiotic was ciprofloxacin (98.1%), followed by co-trimoxazole (97.2%), cefuroxime (91.5%), and nitrofurantoin (91.5%). Furthermore, clinical information received about possible previous treatment and the presence of concomitant diseases was estimated to be available in about one-fifth of urine samples only (on average 21.3% and 21.5%, respectively).

### Strengths and limitations

The high response rate of 65.2% is a notable strength of our study. However, out of the 258 participating laboratories, only 106 performed susceptibility testing for outpatient UTIs. Since no publicly available lists of all outpatient laboratories in Germany exists, it remains unclear whether all laboratories primarily serving outpatients could be reached in the survey, despite using various sources to identify them. Another limitation is that only estimates of the proportion of information accompanying urine samples were requested and no samples were analysed. Beyond that, no further follow-up questions were asked, such as why (recommended) antibiotics were not tested or how the susceptibility tests were technically performed, as we aimed to keep the questionnaire concise to encourage participation. Additionally, information exchange (for example, data transmission and the space allowed for clinical details) and the communication between the laboratory and the outpatient physicians was not explored. Furthermore, the information provided relates only to the testing of *E. coli* from urine cultures.

### Comparison with existing literature

While most laboratories tested for nitrofurantoin, fosfomycin, and pivmecillinam, considerably fewer included trimethoprim and, above all, nitroxoline. The three most frequently tested antibiotics include a fluoroquinolone (ciprofloxacin) and a cephalosporin (cefuroxime), which should only be used as second-line antibiotics or for complicated infections owing to side effects and the increased risk of antibiotic resistance.^12,20^ Our results are comparable with those of Schmiemann *et al* for northern Germany in the study from 2013, when ciprofloxacin (95%) and cefuroxime (93%) were also among the most frequently tested antibiotics, while only 58% of laboratories tested for trimethoprim. No comparative data are available for pivmecillinam, which has been available in Germany since 2016.^14^ Reasons why laboratories may not perform susceptibility testing for trimethoprim may be the testing for co-trimoxazole, the lack of inclusion of trimethoprim in susceptibility testing cards, or the local resistance rates.^21^ Nevertheless, studies suggest that trimethoprim resistance rates in community-acquired UTIs may be overestimated, as routinely collected data typically reflect resistance rates in complicated UTIs, whereas no further diagnostic measures are usually performed in uncomplicated UTIs.^10,11,22^ Only 34% of laboratories performed standard susceptibility testing for nitroxoline, although it has also been included in the German guidelines since 2017 and can be an alternative as it has a low in vitro resistance rate in *E. coli* and retains a good activity even in multidrug-resistant isolates.^23,24^ One main reason for this may be that many laboratories are using automated systems to perform susceptibility testing, which can deliver results in less time and at lower cost.^25^ However, nitroxoline is not included in most standard test panels of automated systems and therefore needs to be tested in addition, at extra costs.^26^ Nitroxoline is also rarely used in other European countries and is not included in the updated European Society of Urology guideline for the treatment of UTIs.^9,27^ Another reason why laboratories may not test for all the antibiotics listed in the guidelines is the fee schedule of the National Association of Statutory Health Insurance Funds, which pays a flat rate for testing. In addition, clinical guidelines do not recommend routine urine culture, so most samples tested in laboratories are likely to be from complicated cases.^12,28^ However, most laboratories do not have the information if the urine is from an uncomplicated or complicated infection, and will therefore likely test antibiotics for both indications. Nevertheless, some first-line antibiotics often can be used in complicated UTIs when susceptibility data are available, and the kidneys are not involved. This may help to reduce reliance on second-line antibiotics, such as ciprofloxacin, to combat antimicrobial resistance.^12,20^

With an average of 13.1 (SD 3.6) different antibiotics, the laboratories in our study tested a wide range of substances, which increases the risk for reporting a large number of susceptible antibiotics to the outpatient physicians without limiting the choice of antibiotic therapies. This might hamper optimal treatment as the performance of susceptibility testing and the reporting have a direct influence on the prescription of antibiotics for UTIs.^15,16,29^ Even more, selective reporting of susceptibility test results can be a powerful tool in antibiotic stewardship and can lead to greater guideline adherence when prescribing antibiotics for UTIs.^16,29^

The laboratories received clinical information on previous treatments and comorbidities in only an estimated one-fifth (on average 21.3% and 21.5%) of urine samples, and information on the type of the urine sample in an estimated three-fifths (63.7%) of samples. However, results of a urine culture need be interpreted in light of the type of urine sample and patients’ clinical presentation, as bacteriuria alone does not constitute a UTI and justify antibiotic therapy.^12,27^ Our study indicates that microbiological laboratories often lack information to select the optimal antibiotics for testing and therapy. The German quality standards MIQ emphasises that meaningful assessment of a urine culture requires clinical information about the patient, and discusses the significance of leukocyturia and the type of urine collection.^13^ Neither the recent German guideline nor the European Association of Urology’s guideline contain any recommendations regarding the specific information that should be provided to a laboratory accompanying a urine sample.^12,28^

### Implications for research and practice

In conclusion, laboratories in Germany tested 13.1 different antibiotics for isolates from urines, but only 26.4% tested for all five antibiotics recommended by German guidelines for uncomplicated UTI. Ideally, laboratories should test all first-line antibiotics recommended in the guidelines. The inclusion of pivmecillinam and nitroxoline in more automated systems could increase the testing frequency of first-line antibiotics in the future. Furthermore, it would be beneficial to prioritise the reporting of first-line antibiotics. Such an approach provides the outpatient physician with an overview of the local resistance situation and could support clinical decision making for future patients. Further highlighting first-line antibiotics in laboratory reports may help to align prescribing practices more closely with current recommendations, reducing the unnecessary use of broad-spectrum antibiotics and contributing to improved antibiotic stewardship.

In addition, laboratories often seem to lack clinical information, which may influence the selection of antibiotics for susceptibility testing. The optimal interpretation of laboratory results also depends on relevant clinical information. Therefore, a more detailed and standardised transfer of clinical information from outpatient physicians to laboratories could improve the quality of antibiotic prescribing and compliance with antimicrobial stewardship.

However, a concise and clearly defined list of specific recommendations on how and what relevant information should be shared with laboratories is still lacking and should be included in the updated guidelines.

## Data

The dataset relied on in this article is available from the corresponding author on reasonable request.

## References

[1] Butler CC, Hawking MKD, Quigley A, McNulty CAM (2015). Incidence, severity, help seeking, and management of uncomplicated urinary tract infection: a population-based survey. Br J Gen Pract.

[2] Yang X, Chen H, Zheng Y (2022). Disease burden and long-term trends of urinary tract infections: a worldwide report. Front Public Health.

[3] Hoffmann T, Peiris R, Mar CD (2020). Natural history of uncomplicated urinary tract infection without antibiotics: a systematic review. Br J Gen Pract.

[4] Schmiemann G, Hoffmann F, Hamprecht A, Jobski K (2022). Patterns and trends of antibacterial treatment in patients with urinary tract infections, 2015–2019: an analysis of health insurance data. BMC Prim Care.

[5] Murray CJL, Ikuta KS, Sharara F (2022). Global burden of bacterial antimicrobial resistance in 2019: a systematic analysis. Lancet.

[6] Schneidewind L, Stangl FP, Dräger DL (2022). [What is the proportion of infectiology in the specialization urology?: a pilot study to underline the significance of antibiotic stewardship in urology]. [Article in German]. Urologie.

[7] Kranz J, Schlager D, Mühlstädt S (2019). [Barriers to guideline adherence: identification of barriers to guideline adherence using a survey on the AWMF S3 guideline epidemiology, diagnosis, treatment, and management of uncomplicated bacterial, community-acquired urinary tract infections in adult patients]. [Article in German]. Urologe A.

[8] Goebel MC, Trautner BW, Grigoryan L (2021). The five Ds of outpatient antibiotic stewardship for urinary tract infections. Clin Microbiol Rev.

[9] Malmros K, Huttner BD, McNulty C (2019). Comparison of antibiotic treatment guidelines for urinary tract infections in 15 European countries: results of an online survey. Int J Antimicrob Agents.

[10] Schmiemann G, Gebhardt K, Hummers E (2018). [DEGAM S3 guideline: burning sensation when urinating]. [Article in German]. https://www.degam.de/files/Inhalte/Leitlinien-Inhalte/Dokumente/DEGAM-S3-Leitlinien/053-001_Brennen%20beim%20Wasserlassen/abgelaufene-leitlinie-brennen-beim-wasserlassen/053-001l_brennen-wasserlassen_langversion_29-08-18-1-1.pdf.

[11] Fünfstück R, Helbig S, Hofmann W (2017). [DGU guideline programme interdisciplinary S3 guideline: epidemiology, diagnosis, treatment, prevention and management of uncomplicated, bacterial, community-acquired urinary tract infections in adult patients]. [Article in German]. https://register.awmf.org/assets/guidelines/043_D_Ges_fuer_Urologie/043-044m2017_S3_Epidemiologie-Diagnostik-Therapie-Praevention-Management-Harnwegsinfektionen-Erwachsene_2024-02.pdf.

[12] Deutsche Gesellschaft für Urologie e.V (2024). [S3 guideline: epidemiology, diagnosis, treatment, prevention and management of uncomplicated, bacterial, community-acquired urinary tract infections in adult patients, update]. [Article in German]. https://register.awmf.org/de/leitlinien/detail/043-044.

[13] Schubert S, Fünfstück R, Gatermann S (2020). [MiQ 02: urinary tract infections: quality standards in microbiological and infectious disease diagnostics]. [Book in German].

[14] Schmiemann G, Noll J, Hoffmann F (2016). [Resistance testing for urinary tract infections. a barrier to guideline implementation]. [Article in German]. Urologe A.

[15] Langford BJ, Daneman N, Diong C (2021). Antibiotic susceptibility reporting and association with antibiotic prescribing: a cohort study. Clin Microbiol Infect.

[16] Simon M, Fougnot S, De Monchy P (2023). Impact of selective reporting of antibiotic susceptibility testing results for urinary tract infections in the outpatient setting: a prospective controlled before-after intervention study. Clin Microbiol Infect.

[17] Schuster A, Tigges P, Grune J (2023). GPs’ perspective on a multimodal intervention to enhance guideline-adherence in uncomplicated urinary tract infections: a qualitative process evaluation of the multicentric RedAres cluster-randomised controlled trial. Antibiotics (Basel).

[18] Schmiemann G, Greser A, Maun A (2023). Effects of a multimodal intervention in primary care to reduce second line antibiotic prescriptions for urinary tract infections in women: parallel, cluster randomised, controlled trial. BMJ.

[19] Flores-Mireles AL, Walker JN, Caparon M, Hultgren SJ (2015). Urinary tract infections: epidemiology, mechanisms of infection and treatment options. Nat Rev Microbiol.

[20] Stapleton AE, Wagenlehner FME, Mulgirigama A, Twynholm M (2020). Escherichia coli resistance to fluoroquinolones in community-acquired uncomplicated urinary tract infection in women: a systematic review. Antimicrob Agents Chemother.

[21] Stoltidis-Claus C, Rosenberger KD, Mandraka F (2023). Antimicrobial resistance of clinical Enterobacterales isolates from urine samples, Germany, 2016 to 2021. Euro Surveill.

[22] Klingeberg A, Noll I, Willrich N (2018). Antibiotic-resistant E. coli in uncomplicated community-acquired urinary tract infection. Dtsch Arztebl Int.

[23] Fuchs F, Hamprecht A (2019). Susceptibility of carbapenemase-producing Enterobacterales (CPE) to nitroxoline. J Antimicrob Chemother.

[24] Plambeck L, Fuchs F, Sattler J, Hamprecht A (2022). *In vitro* activity of mecillinam, temocillin and nitroxoline against MDR Enterobacterales. JAC Antimicrob Resist.

[25] Gajic I, Kabic J, Kekic D (2022). Antimicrobial susceptibility testing: a comprehensive review of currently used methods. Antibiotics (Basel).

[26] Schaumburg F, Gatermann SG, Becker K (2018). Guidelines for interpretation required. Dtsch Arztebl Int.

[27] Kranz J, Bartoletti R, Bruyère F (2024). European Association of Urology guidelines on urological infections: summary of the 2024 guidelines. Eur Urol.

[28] Bonkat G, Bartoletti R, Bruyère F (2024). EAU guidelines on urological infections.

[29] Bourdellon L, Thilly N, Fougnot S (2017). Impact of selective reporting of antibiotic susceptibility test results on the appropriateness of antibiotics chosen by French general practitioners in urinary tract infections: a randomised controlled case-vignette study. Int J Antimicrob Agents.

